# Locally advanced pancreatic cancer successfully treated by distal pancreatectomy with celiac axis resection (DP-CAR) after S-1 with radiation therapy followed by gemcitabine/nab-paclitaxel therapy: a case report

**DOI:** 10.1186/s40792-017-0290-6

**Published:** 2017-01-18

**Authors:** Kyohei Ariake, Fuyuhiko Motoi, Masamichi Mizuma, Keigo Murakami, Tatsuyuki Takadate, Hideo Ohtsuka, Koji Fukase, Kunihiro Masuda, Hiroki Hayashi, Kei Nakagawa, Naoaki Sakata, Takanori Morikawa, Shimpei Maeda, Takeshi Naitoh, Shinichi Egawa, Michiaki Unno

**Affiliations:** 1 Department of Surgery, Tohoku University Graduate School of Medicine, Seiryo-machi, Aoba-ku, Sendai, 980-8574 Japan; 2 Department of Pathology, Tohoku University, 1-1, Seiryocho, Aobaku, Sendai, Japan; 3 Division of International Cooperation for Disaster Medicine, Tohoku University, 1-1, Seiryocho, Aobaku, Sendai, Japan

**Keywords:** Pancreatic cancer, DP-CAR, Chemoradiation therapy, Gemcitabine, Nab-paclitaxel, Locally advanced

## Abstract

**Background:**

The prognosis for pancreatic cancer remains dismal because many patients are diagnosed with unresectable cancer at the initial diagnosis. Recently, conversion surgery was reported as an effective treatment for initially unresectable pancreatic cancer with a favorable response to non-surgical treatment lasting over 240 days. Here, we describe a case of locally advanced pancreatic cancer (LAPC) successfully resected after treatment with S-1 and radiation followed by gemcitabine/nab-paclitaxel therapy.

**Case presentation:**

A 73-year-old man with LAPC was referred to our hospital. Computed tomography findings revealed a 2.5-cm mass in the pancreatic body that had invaded the celiac artery, common hepatic artery, and splenic artery. Superior mesenteric artery (SMA) encasement was not observed, but tumor abutment over 180° with the main tumor was detected. Staging laparoscopy showed no findings of distant metastasis, and washing cytology revealed no malignancy. He was diagnosed with unresectable pancreatic cancer. Treatment with S-1 with radiation therapy followed by gemcitabine with nab-paclitaxel was performed. Six months after the initial treatment, the tumor size had decreased to 1.2 cm, and encasement of the main artery was diminished. Though abutment to the main artery, including the SMA, was still detected, distal pancreatectomy with celiac artery resection was performed. The histopathological findings around the celiac artery revealed fibrous changes with an Evans classification of grade IIb. There was no residual cancer at the periphery; thus, R0 resection was achieved. The patient has been healthy and without recurrence for more than 12 months since the initial treatment.

**Conclusions:**

Gemcitabine/nab-paclitaxel therapy revealed high response rate for metastasic pancreatic cancer (PC), but the effect for LAPC proposing conversion surgery was not well discussed. In this case, we achieve R0 resection combined with chemoradiation therapy and gemcitabine/nab-paclitaxel therapy. This regimen was also effective for LAPC and may be used to increase the population of conversion surgery by its high response rate.

## Background

The prognosis for pancreatic cancer (PC) remains dismal because many patients receive an initial diagnosis of unresectable (UR) PC [1]. Among these patients, 30–40% are considered to have locally advanced pancreatic cancer (LAPC).

The standard treatment for LAPC has been chemotherapy or chemoradiation therapy [2–4]. Recently, powerful regimens, such as FOLFIRINOX or gemcitabine with nab-paclitaxel, have demonstrated a high response rate for metastatic PC [5–8]. In particular, FOLFIRINOX has been used as a neoadjuvant treatment for LAPC, resulting in a high conversion rate to surgical resection by improving tumor shrinkage [9, 10]. Only the curative treatment for PC is surgical resection; therefore, treatment resulting for conversion surgery was an effective treatment strategy. The appropriate length before surgical treatment was now controversial. Satoi et al. revealed that surgical resection for definitively selected patients who responded to non-surgical anti-cancer treatment for more than 240 days could achieve long-term survival for initially unresectable PCs [11]. However, the report did not include the recent effective regimen such as FOLFIRINOX and Gemcitabine/Nab-Paclitaxel.

Here, we present a case of successful achieved conversion surgery for initially unresectable PC after treatment with chemoradiotherapy followed by chemotherapy with gemcitabine/nab-paclitaxel for under 200-day treatment durations.

## Case presentation

**Fig. 1:**
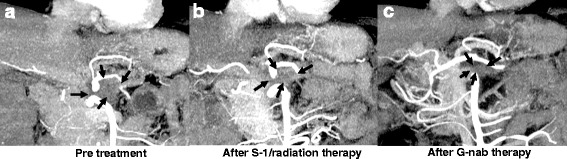
Coronal computed tomography images. **a** Before chemoradiation. **b** After chemoradiation. **c** After gemcitabine with nab-paclitaxel therapy. There are no significant differences in tumor volume between **a** and **b**, but **c** demonstrates tumor shrinkage to 1.2 cm

**Fig. 2:**
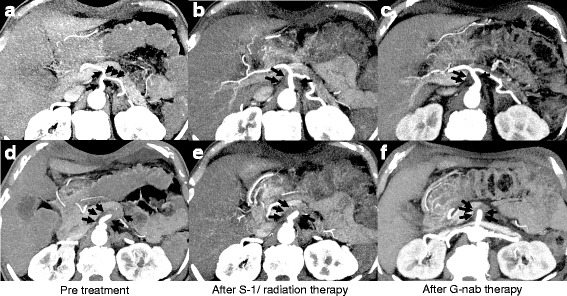
Axial computed tomography images showing the celiac artery, common hepatic artery, splenic artery, and superior mesenteric artery. **a**, **d** Before chemoradiation therapy. **b**, **e** After chemoradiation therapy. **c** ***,*** **f** After gemcitabine with nab-paclitaxel therapy. **a** Encasement of the CA, CHA, and SA by direct invasion of tumor (*arrows*). **b** A reduction of encasement in the CHA and SA, but abutment to the SMA is still detected (*arrows*). **c** Suspected tumor abutment to the SMA (*arrows*). In **f**, tumor volume is decreased compared to that in **d** and **e**, but tumor abutment to the SMA was detected in all figures (*arrows*)

A 73-year-old man presented to his local hospital with a 3-month history of inappetence and body weight loss. Abdominal ultrasonography revealed pancreatic body cancer, and he was referred to our hospital. He had type 2 diabetes mellitus and a history of appendectomy performed when he was 20 years of age. His family history included that his uncle has a history of gastric cancer. Laboratory data revealed a high level of hemoglobin A1c (8.1%), but tumor markers, including carcinoembryonic antigen (2.1 ng/mL), cancer antigen-19-9 (15.4 U/mL), Dupan-2 (29 U/mL), and Span-1 (11.4 U/mL), were all within normal limits. Computed tomography (CT) findings revealed a 2.5-cm mass in the pancreatic body (Fig. 1a). The celiac artery (CA), common hepatic artery (CHA), and splenic artery (SA) showed encasement by direct tumor invasion (Fig. 2a). There was no encasement of the superior mesenteric artery (SMA), but abutment of over 180° with the main tumor was seen (Fig. 2d). Magnetic resonance cholangiopancreatography revealed stenosis of the main pancreatic ducts with upstream dilatation of the pancreatic duct. Endoscopic retrograde cholangiopancreatography imaging also demonstrated pancreatic duct strictures near the pancreatic body. Endoscopic ultrasonography-guided fine needle aspiration cytology was performed, and pathological findings revealed a pancreatic adenocarcinoma. Positron emission tomography (PET) findings did not show the possibility of distant metastasis, and the maximum standard uptake value of the main tumor was 2.5. Staging laparoscopy showed no findings of peritoneal and/or liver metastasis. Additionally, washing cytology did not show malignancy. From these findings, we diagnosed the patient with unresectable locally advanced pancreatic cancer (UR LAPC).

Based on these findings, S-1 with radiation therapy was performed. Five days of S-1 intake (80 mg/m^2^/day) with radiation therapy (2 Gy/day) was performed per week. This treatment continued for 5 weeks, and the total radiation amount was 50 Gy. During this treatment, the patient experienced no significant adverse effects. After chemoradiation, CT findings showed that the encasement of the CA, CHA, and SA was released, but abutment to the SMA was still detected (Figs. 1a and 2b, e), so he was diagnosed as still having LAPC. Therefore, chemotherapy with gemcitabine and nab-paclitaxel followed.

At first, the standard regimen (i.e., days 1, 8, and 15: injection of gemcitabine (1000 mg/m^2^) and nab-paclitaxel (125 mg/m^2^) every 4 weeks) was proposed. However, because grade 3 thrombocytopenia was observed, biweekly chemotherapy was performed 12 times, and other side effects were not observed. After the treatments, the tumor size decreased to 1.2 cm (Fig. 1c), but abutment to the CA, CHA, SA, and SMA was still detected (Fig. 2c, f). PET findings did not show the possibility of distant metastasis, and the maximum standard uptake value for the main tumor was 2.4. Tumor markers, including carcinoembryonic antigen (2.9 ng/mL), cancer antigen-19-9 (12.4 U/mL), Dupan-2 (25.0 U/mL), and Span-1 (11.3 U/mL) were still all within normal limits.

We initially proposed embolization of the CHA and the left gastric artery before surgical treatment. However, the CHA was too short to be coiled safely and had a risk of embolization for the proper hepatic artery. Therefore, we only embolized the left gastric artery, expecting increased blood perfusion from the right gastric artery, and right gastroepiploic artery to avoid acute ischemic disease of the stomach after celiac axis resection. Six days after blocking the left gastric artery, surgical treatment was performed.

**Fig. 3:**
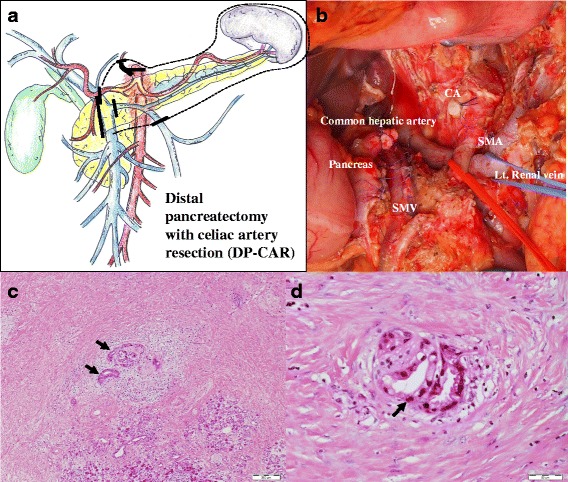
Operative and histopathological findings. **a** Schematic diagram of this surgical treatment. The *bar* shows the resection lines. **b** Intraoperative image obtained after tumor resection. **c**, **d** The histopathological findings around celiac artery. About 80% of cancer cells were diminished with a desmoplastic stroma. *Arrows* show residual degenerated cancer cells with perineural invasion

Intraoperative findings showed neither peritoneal nor liver metastasis. Intraoperative washing cytology revealed no findings of malignancy. A 2.0-cm tumor existed at the pancreatic body. It had invaded to the splenic vein, but far from the portal vein. Though abutment to SMA was still detected around the SMA by CT findings, intraoperative findings showed fibrous changes around the right half of the SMA and it could be easily separated from SMA. From these findings, we think that two thirds resection of the nerve plexus including fibrous change around the SMA was enough to achieve R0 resection. Based on these findings, we performed distal pancreatectomy with celiac axis resection with two thirds of the nerve plexus resection around the SMA (Fig. 3a, b). The total operation time was 438 min, and blood loss was 1239 mL.

Histopathological findings showed that a 0.8 × 0.5 cm, moderately differentiated tubular adenocarcinoma with marked degeneration, was present at the pancreatic body; 50–60% of the tumor was changed to fibrous tissue, defined as grade IIb in the Evans classification (Fig. 3c, d). The UICC TNM classification (7th edition) was defined as T1N0M0, stage IA. Including the nerve plexus around the SMA, there were no residual cancer cells in the dissected margin and/or the pancreatic resection margin. Therefore, we achieved R0 resection.

After surgical treatment, there were grade B complications of the pancreatic fistula, but they were controlled by percutaneous drainage not under general anesthesia (Clavien-Dindo IIIa). The patient was discharged from the hospital 52 days after surgical treatment. He has now been treated with S-1 as adjuvant chemotherapy and has done well without recurrence for more than 12 months from the initial treatment.

## Discussion

The recommended first-line treatment for LAPC is chemotherapy or chemoradiation therapy [2–4]. In this case, we initially planned to administer radiation therapy to the nerve plexus around the SMA combined with S-1 therapy, expecting a highly favorable response of the LAPC [12, 13].However, abutment to the SMA was not diminished after chemoradiation therapy, and the patient was still diagnosed with UR LAPC. Thus, gemcitabine/nab-paclitaxel therapy was selected as the second-line treatment, considering that it has a high response rate for metastatic PC (approximately about 58.8% for Japanese phase I/II cohort) expecting the down-staging of the tumor [5]. However, after this round of chemotherapy, tumor abutment around the SMA was still not diminished. In this case, the most difficult problems for conversion surgery were to determine the effect of anti-cancer therapy and when to perform surgical treatment. The past literature demonstrated that it can be difficult to evaluate local tumor progression accurately by radiological evaluation after chemotherapy and/or chemoradiation therapy [14]. And the other studies have shown that tumor markers or PET findings are sometimes helpful in estimating the efficacy of anti-cancer therapy [15, 16].However, in this case, tumor markers were not increased and PET findings were normal before the first-line treatment. Therefore, we could diagnose the tumor status only based on the CT or MRI findings. We usually decided to perform surgical treatment on the basis of four findings: (1) the tumor itself demonstrated relative shrinkage, (2) there were no obvious metastatic sites, (3) tumor marker levels were all normal range, and (4) PET findings were in the relatively lower range. In this case, we decided the operation because marked tumor regression was observed and maintained for several months after chemotherapy, although the finding around SMA was not completely diminished. An accurate diagnosis before surgical treatment remains difficult and that the criteria for surgical resection are now controversial. If there is a possibility for conversion surgery, proposing surgical treatments and making a diagnosis based on intraoperative findings are necessary for LAPC.

Conversion surgery for LAPC was the only cure for LAPC. A previous study recommended conversion surgery for patients who responded to non-surgical anti-cancer treatment for more than 240 days [11]. In that study, most patients were treated with gemcitabine-based regimens such as gemcitabine monotherapy or gemcitabine/S-1 therapy. Recently, FOLFIRINOX or gemcitabine with nab-paclitaxel therapy has shown a high response rate for UR PC [5–8, 17]. FOLFIRINOX has also been used as a treatment for LAPC and resulting in a high conversion rate to surgical resection [18]. Although gemcitabine/nab-paclitaxel therapy also demonstrated a high response rate for metastatic PC [5], the evidence for its use in LAPC is now controversial. In this case, we successfully achieved R0 resection for UR LAPC by using gemcitabine/nab-paclitaxel therapy after S-1 radiation therapy under 200-day treatment. Our case suggested that this regimen was also effective for LAPC and had the possibility to reduce the length of treatment. In the future, this strong regimen might be proposed as a good strategy for conversion surgery, and could result in the increase in the number of patients who could benefit from conversion surgery.

## Conclusions

We achieved R0 resection with S-1/radiation therapy followed by gemcitabine/nab-paclitaxel therapy. This regimen may be used to increase the population who could benefit from conversion surgery.

## Abbreviations

CA: Celiac artery
CHA: Common hepatic artery
CT: Computed tomography
DP-CAR: Distal pancreatectomy with celiac axis resection
LAPC: Locally advanced pancreatic cancer
PC: Pancreatic cancer
PET: Positron emission tomography
SA: Splenic artery
SMA: Superior mesenteric artery
UR: Unresectable
